# Supplementary material for: Identification of photoperiod-induced specific miRNAs in the adrenal glands of Sunite sheep (Ovis aries)
Source: Front Vet Sci. 2022 Jul 22;9:888207. doi: 10.3389/fvets.2022.888207 (PMC9354845; doi:10.3389/fvets.2022.888207)
Supplement: Supplementary file 1 [file Data_Sheet_1.ZIP › Supplementary file/miRDeep2 file.zip!/miRDeep2 file/Know_Structure/LP42A1/oar-mir-154b.pdf]

miRBase precursor : oar-mir-154b
Total read count : 1344
oar-miR-154b-5p read count : 1270
oar-miR-154b-3p read count : 74
remaining reads : 0

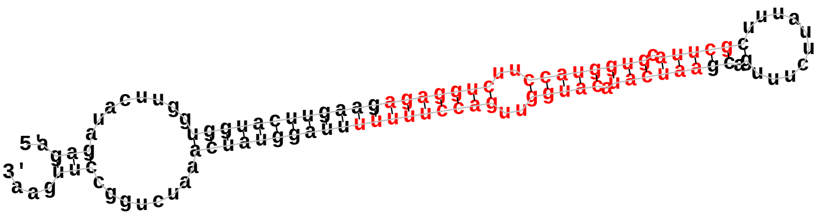

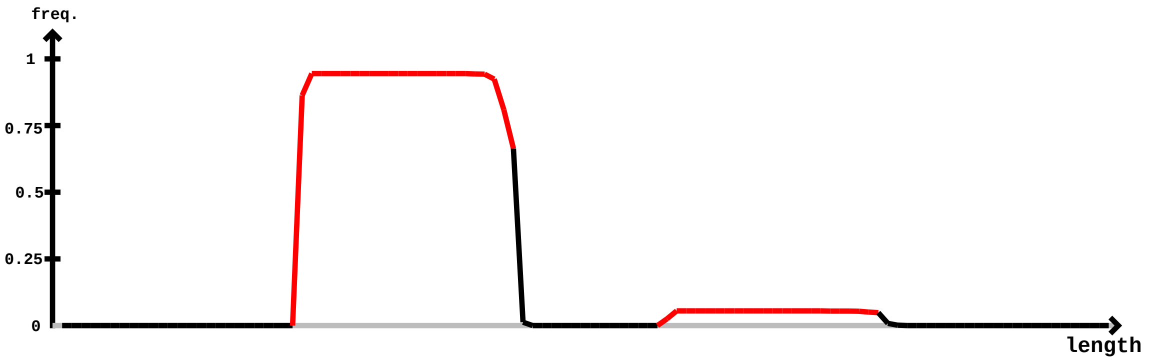


```
                                                                           oar-miR-154b-3p
                        oar-miR-154b-5p
5'- agagauacuuggugguacuugaagagaggucuuccauggugcauucgcuuuauucuuugacgaaucauacaugguugaccuuuuuuuagguaucaaaucuggccuugaa  -3'   exp
    .(((........((((((((((((((((((..((((((((.((((((..........).)))))))).))))))..)))))))))))))))))))........)))...    reads     mm      sample
    ........................agaggucuuccauggugc...................................................................    2         0       seq
    ........................agaggucuuccauggugca..................................................................    1         0       seq
    ........................agaggucuuccauggugcau.................................................................    25        0       seq
    ........................agaggucuuccauggugcauu................................................................    146       0       seq
    ........................agaggucuuccauggugcauuc...............................................................    188       0       seq
    ........................agaggucuuccauggugcauucg..............................................................    784       0       seq
    ........................agaggucuuccauggugcauucgc.............................................................    14        0       seq
    .........................gaggucuuccauggugcauu................................................................    8         0       seq
    .........................gaggucuuccauggugcauuc...............................................................    8         0       seq
    .........................gaggucuuccauggugcauucg..............................................................    91        0       seq
    .........................gaggucuuccauggugcauucgc.............................................................    3         0       seq
    .............................................................aaucauacaugguugaU...............................    1         1       seq
    .............................................................aaucauacaugguugaccuu............................    1         0       seq
    .............................................................aaucauacaugguugaccuuu...........................    4         0       seq
    .............................................................aaucauacaugguugaccuuuu..........................    3         0       seq
    .............................................................aaucauacaugguugaccuuuuu.........................    24        0       seq
    .............................................................aaucauacaugguugaccuuuuuu........................    1         0       seq
    ..............................................................aucauacaugguugaccuuuuu.........................    30        0       seq
    ..............................................................aucauacaugguugaccuuuuuu........................    8         0       seq
    ..............................................................aucauacaugguugaccuuuuuuu.......................    2         0       seq
```
